# Societal consequences of IPS implementation in Norway 2012–2019: study protocol for the IPSRON effectiveness study

**DOI:** 10.1136/bmjopen-2025-102179

**Published:** 2026-03-06

**Authors:** Arnstein Mykletun, Nils Abel Aars, Thomas Lorentzen, Miles Rinaldi, Elisabeth Sandtorv, David McDaid, Cathrine Fredriksen Moe, A-La Park, Eoin Killackey, B Brinchmann

**Affiliations:** 1 Centre for Population Health, Haukeland University Hospital, Bergen, Hordaland, Norway; 2 Centre for Health and Work, Nordlands Hospital Trust, Bodø, Nordland, Norway; 3 Department of Community Medicine, UiT The Arctic University of Norway, Tromsø, Norway; 4 Division for Health Sciences, Norwegian Institute of Public Health, Oslo, Norway; 5 Department of Sociology, University of Bergen, Bergen, Vestland, Norway; 6 Centre for Research and Education in Forensic Psychiatry, Haukeland University Hospital, Bergen, Norway; 7 Care Policy and Evaluation Centre, Department of Health Policy, LSE, London, UK; 8 Faculty of Nursing and Health Sciences, Nord universitet, Bodø, Nordland, Norway; 9 Orygen The National Centre of Excellence in Youth Mental Health, Parkville, Victoria, Australia; 10 The University of Melbourne Centre for Youth Mental Health, Parkville, Victoria, Australia

**Keywords:** EPIDEMIOLOGY, EPIDEMIOLOGIC STUDIES, Health Services, MENTAL HEALTH, Adult psychiatry, PUBLIC HEALTH

## Abstract

**Introduction:**

Individuals experiencing moderate to severe mental illness have low rates of workforce inclusion, with a consequence of high welfare dependency, affecting both societal costs and health. Individual Placement and Support (IPS) is an approach to supported employment where the goal is to help people obtain jobs on the open rather than sheltered labour markets. Despite multiple randomised controlled trials (RCTs) indicating that the IPS model enables employment better than treatment as usual, with widespread adoption in some jurisdictions, the broader impacts of this large-scale implementation on mental health, quality of life and social functioning remain unknown.

**Methods and analysis:**

Between 2012 and 2019, Norway introduced IPS through both local and national government projects. This study assesses the social and economic benefits of the implementation of IPS using Norwegian registry data, focusing on 18–45-year-old people receiving specialist mental healthcare, and who did not have steady employment at treatment start. Instead of assessing IPS efficacy in an RCT design, we use a naturalistic study design, evaluating IPS effectiveness by comparing aggregate population-level outcomes over time between areas where IPS was not available.

In work package (WP) 1, we mapped the availability and implementation of IPS across Norway. This involved analysing information on funding, resource and capacity levels to understand how IPS had been rolled out across the country. While completed, we include a description of WP1 here, as it informs WP2 and WP3. WP2 is an effectiveness evaluation investigating the population-level outcomes of implementing IPS, focusing on health, mortality, quality of life and social functioning. Finally, in WP3, we assess the financial implications of implementing IPS from a public purse perspective, synthesising data on resource use and costs of implementation with data from WP2.

Overall, we will examine the societal effects of IPS implementation on employment, welfare dependency, mental healthcare use, emergency care visits, self-harm and suicide, general mortality, crime and victimisation. Emphasis will be on long-term outcomes, and we will model the economic consequences of IPS. This study aims to inform policy making and strategies for implementing IPS at scale.

**Ethics and dissemination:**

This is an effectiveness study using registry data. The Regional Committee for Medical Research Ethics Northern Norway, REK North has approved the use of registry data without informed consent for this project (approval number 134553).

The findings will be disseminated both in academic peer-reviewed journals, directly to informants in WP1, to the public through media and the project website, and at relevant conferences and seminars for specific relevant target groups.

**Trial registration number:**

Not applicable

**STRENGTHS AND LIMITATIONS OF THIS STUDY**

- This study leverages a national staggered rollout of Individual Placement and Support (IPS) in Norway, allowing for a robust difference-in-differences design to assess population-level effectiveness beyond controlled experimental conditions.
- By using high-quality, nationwide administrative registries covering health, employment, welfare, crime and mortality, the study ensures minimal attrition and enhances the validity and reliability of outcomes.
- Relying on local site reports to capture IPS implementation intensity introduces a risk of recall or reporting bias.
- We have limited knowledge of interventions and initiatives in the control group. To the extent there are IPS-like activities in the control group, this would introduce bias in favour of the nil hypothesis.
- As an observational (non-randomised) design, residual confounding may persist despite efforts to adjust for baseline differences.

## Introduction

 Work is central to well-being, providing income, identity, structure and social connection.^1 2^ Employment is linked to lower rates of loneliness^3^ and suicide,^4^ and extensive evidence shows better physical and mental health among employed people across time, settings and socioeconomic groups.^15^,^7^ Despite advances in treatment, most people with severe mental illness (SMI) remain excluded from the workforce.^1^,^5^ Employment typically declines with continued service contact,^8^ though many regard employment as central to recovery and an important treatment goal.^9^,^13^

Work has long been regarded as integral to mental health treatment, contributing to purpose, self-efficacy and belonging.^14^,^16^ During the 20th century, work moved from being viewed as a form of therapy to being considered a human right. Over the last 40 years, vocational rehabilitation has evolved into two main models: traditional vocational rehabilitation (‘train and place’) and supported employment (‘place and train’), such as Individual Placement and Support (IPS). Developed in the 1990s in the USA, IPS helps people with serious mental illness gain employment through a rapid ‘place-then-support’ approach,^17^ and the model is internationally recognised as the most effective evidence-based practice for helping people living with SMI obtain competitive employment,^18 19^ with demonstrated superiority over all other forms of vocational rehabilitation. It is based on eight evidence-based principles, is manualised^20^ and has a fidelity scale^21^ to assess whether it is being implemented as intended. Meta-analyses of IPS randomised controlled trials (RCTs) consistently find employment rates to be more than double compared with other vocational rehabilitation approaches regardless of diagnostic, clinical, functional and personal characteristics.^22 23^ Furthermore, systematic reviews of its cost-effectiveness find a strong economic case for implementation.^24^

Increasingly, IPS is being recommended within national mental health treatment guidelines for people who wish to gain and retain employment.^25^,^27^ Policy makers see strengths of IPS being the strict fidelity adherence and the continuous monitoring of the implementation rules.^28^ Internationally, IPS is available in over 20 countries,^29^ many of which are undertaking expansions supported through national strategies and targeted funding.^30^,^32^

Mental health conditions remain the leading contributor to years lived with disability (YLDs) for young people aged 10–24 and the second highest contributor to YLDs for all age groups in high-income countries.^33^ The increase in disability rates among young people is particularly concerning, and the increase among young people is predominantly for mental health conditions.^34^ In Norway, mental health conditions are now the main reason for claiming disability pension—surpassing musculoskeletal disorders in 2011.^35^ During the same period, the prevalence of disability among young people due to mental health conditions has increased by almost 50%,^34^ while disability among older people has remained largely stable. This raises significant social, political and economic concerns, as they contribute to a widening employment gap.

RCTs have demonstrated IPS effectiveness at the individual level in countries with different labour markets, welfare systems and economic conditions, with little effect on outcomes.^22 36^ A recent Norwegian study found a significant, positive, causal effect on societal-level employment outcomes for young adults in a municipality where IPS was implemented compared with municipalities where it was not.^37^ In the Netherlands, a national evaluation of IPS^38^ found similar results comparing outcomes for disability benefit recipients with mental health conditions receiving IPS to a control group receiving traditional vocational rehabilitation services.^39^ While this emerging evidence for a societal impact is encouraging, whether the IPS expansions can produce population-level health and economic benefits (and implications for societal well-being) remains unknown.

Crime is another relevant societal outcome, given its substantial social costs.^40^ People living with mental health conditions are more likely to be victims of crime and have higher rates of contact with the criminal justice system, partly due to poverty, social exclusion and increased risk of drug and alcohol dependency.^41 42^ Whether supported employment programmes such as IPS influence crime at the societal level remains unknown, and will be explored in the current study. The IPS method is costly, and the implementation of IPS services implies increasing the initial outlay to society in expenses for people with mental health conditions. Our previous systematic review^24^ indicates that IPS can be cost-effective from a health system perspective, but these data mainly cover short time periods of under 2 years.

The IPS implementation in the Rest of Norway (IPSRON) study aims to address these knowledge gaps in an effectiveness trial where we will ascertain the societal footprint of IPS implementation at a national level in Norway.

## Methods and analyses

### Study design and setting

The IPSRON project is an effectiveness study using registry data to study the effect of IPS implementation in Norway between 2012 and 2019, as the pandemic hampered further rollout of IPS from 2020.^43^ The project commenced in 2019 and will continue to 2027. It will examine the effect of implementing IPS on society at large, thus studying the effectiveness (as opposed to the efficacy) of IPS. This is the same approach as our study examining the effectiveness of implementing IPS in one Norwegian municipality compared with some control municipalities.^37^ The research project is led by the Centre for Work and Mental Health, Nordland Hospital Trust, Bodø, Norway.

Naturalistic trials, or *natural experiments*, generally involve no active intervention by the researcher. Instead, they rely on observations of phenomena occurring within real-world contexts. Unlike RCTs, natural experiments are not bound by the same temporal, practical or ethical constraints. This allows for analyses over longer timeframes, the inclusion of additional outcome measures and the use of larger (in some cases whole-population) datasets. These features can produce markedly different findings on efficacy and effectiveness. For example, studies comparing long-acting injectable antipsychotics (LAIs) with oral antipsychotics have shown that observational studies often report greater benefits of LAIs than do RCTs.^44^ In Norway, variation in service provision (despite national guidelines and a unified public health and welfare system) combined with substantial geographical diversity creates a unique natural laboratory for research. When coupled with Norway’s world-class health and social registries, this variation enables robust causal analyses of real-world treatment effects on long-term outcomes, generating evidence that can directly inform practice and policy.^45 46^ In the present study, we exploit regional differences in the timing of IPS implementation as a natural experiment to evaluate its effectiveness at the population level.

### Setting

Norway is a high-income nation with low unemployment rates, high job security and more evenly distributed income and wealth than most OECD (Organisation for Economic Co-operation and Development) countries,^47^ indicated by a Gini coefficient of 0.27 (perfect equality would have a value of 0 while 100 would be perfectly unequal).^48^ A key feature of the Norwegian welfare system is social security for the population. This includes free access to higher education, a social safety net for people with reduced health and income, and a universal publicly financed healthcare system.

The two decades following the publication of the World Health Report 2001, which focused on mental health, have seen many high-income countries implement different mental health system reforms. In Norway, reforms between 1998 and 2008^49^ aimed to increase autonomy, reduce dependency and therefore empower people living with mental health conditions. The balance between inpatient and community-based services was changed, with the number of hospital beds dramatically reduced. People were able to receive care and support from more rural psychiatric units rather than centralised large hospitals.^50^ However, this reform was unsuccessful in terms of promoting employment. The continued high number of young people in Norway receiving disability benefits signifies that, almost a decade later, we still have not translated increased access and support from local psychiatric units to greater employment.

### Mental healthcare

In Norway, the state is responsible for hospital and specialist healthcare services, while the municipalities are responsible for primary care and public health which include access to general practitioners.^35^ Specialist mental healthcare is organised through four regional health authorities that organise hospital care and district psychiatric centres. Approximately 250 000 people receive services from specialised mental healthcare annually. Most people are treated in outpatient clinics, assertive community treatment (ACT) teams or flexible ACT teams.^51^ In 2019, mental disorders and substance use disorders accounted for 20.7% of total health spending. For those aged 15–49, mental disorders and substance use disorders accounted for 46.0% of total health spending.^52^

### Work and welfare

The government-funded public employment service (Norwegian Welfare and Labour Administration (NAV)) provides all employment and welfare services in Norway and is thus a ‘one-stop shop’ in line with the international trend of transforming welfare systems.^53^ NAV’s main objectives include having more people employed and thus requiring fewer welfare benefits, the simplification and tailoring of services to meet individual needs and providing comprehensive and efficient labour and welfare support. Studies indicate that between 4 and 13% of people experiencing schizophrenia are working in Norway.^54^,^56^

### Individual Placement and Support in Norway

Since 1997, Norwegian health policy has prioritised the employment of people with mental health conditions,^49^ and in 2007, the Ministry of Labour and Social Inclusion and the Ministry of Health and Care Services jointly published a national strategic plan for work and mental health.^32^ This policy framework highlighted IPS and recognised the need for coordinated support and collaboration from health and social services and NAV to support individuals with mental health conditions to be able to work. In Norway, IPS therefore evolved from a mental health service model to one coordinated through NAV, supported by national resource centres, fidelity measures and national professional guidelines. Currently, around 100 services are operating and a nationwide rollout for young adults was launched in 2021.^57^,^59^ In 2012, a Norwegian IPS RCT for people with mental health conditions was commissioned which found IPS to be more effective than high-quality usual care for both vocational and non-vocational outcomes.^60^ Resources for the implementation of IPS were initially provided through earmarked funding from the Norwegian Directorate of Health (2012–2016) to six to eight pilot sites, covering staff positions, training and operational costs. Since then, resources have gradually expanded and changed. From 2017, funding and responsibility were transferred to NAV, supported by the Health Directorate and the Norwegian Resource Centre for Community Mental Health. National and regional resource centres, IPS advisors, training programmes, fidelity reviews and implementation toolkits were established to ensure quality and sustainability. These developments and resource changes are described in more detail in Moe *et al*,^57^ Moe *et al*,^61^ and Sveinsdottir *et al*.^62^ Some indications of programme drift have been reported: participants have expressed concern that IPS in Norway may deviate from the original evidence-based practice (Moe *et al*^57^) and similar patterns have been observed in other Nordic countries.^63^ A recent Norwegian status review of IPS, commissioned by the Directorate of Health, indicates that weakened integration between NAV and health services threatens a core element of IPS—integrated vocational and psychiatric rehabilitation—critical for individuals with severe and complex mental health conditions.^64^ While cultural and contextual adaptations of IPS are often necessary, research emphasises that such modifications should not compromise its core principles,^65^ which may be relevant in the Norwegian context.

### Work packages, data sources and methods

**Figure 1:**
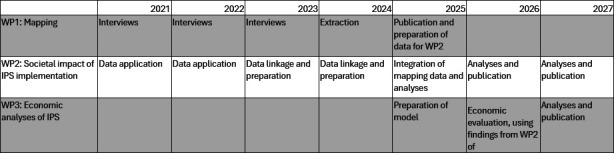
Schematic overview of the timeline for the IPSRON project and work packages. IPS, Individual Placement and Support; IPSRON, IPS implementation in the Rest of Norway; WP, work package.

The three work packages (WPs) in the IPSRON project are inter-related but have distinct data sources and methods and are therefore presented separately. A schematic overview of the timeline for the WPs is presented in figure 1. A premise for the study is WP1, which was initiated in 2021 and completed in 2025. As it pertains to the work in WP2 and WP3, a description of WP1 is included in the section below, with further details described in a previous publication from this project.^58^ The data collected as part of WP1 will become available for linkage with the other data sources in the project in late 2025. Application for registry data to support analysis in WP2 was initiated in 2021–2022. In 2023–2024, the different data were received by the project from its respective registries, and data cleaning and linkage were initiated. In 2025, these data will be linked to the data provided by WP1, at which point all the necessary factors are in place to conduct analyses of the main outcomes of WP2. The economic evaluations of IPS will be informed by results from WP2.

As we make use of complete population data rather than survey samples, this minimises the need for sample selection adjustments through matching procedures. Instead, we will carefully examine whether the identifying assumptions for difference-in-differences are plausible in our context. Specifically, we will:

- Test the parallel trends assumption by comparing pre-intervention trends in outcomes across treatment and comparison areas.
- Assess the comparability of treatment and comparison groups with respect to demographic and socioeconomic characteristics prior to implementation.
- Use a staggered difference-in-differences design (DID), which has been shown to handle variation in treatment timing more effectively, and which we have previously applied with success in related work.^66^

Because Norwegian administrative registries capture nearly all specialist mental health service use and welfare data at the individual level, the study is not susceptible to significant dropout rates or missing data commonly seen in prospective studies. If substantial deviations from parallel trends are observed, we will conduct sensitivity analyses to explore the robustness of our findings, for example, by restricting to more comparable subgroups or using alternative model specifications.

#### WP1: mapping of the implementation of Individual Placement and Support in Norway between 2012 and 2019

The aim of WP1 was to document the implementation of IPS services across Norway between 2012 and 2019, to inform the work in WP2 and WP3. WP1 had commenced at the time of submission of this protocol and has now been published.^58^ In short, two members of staff at the Centre for Work and Mental Health conducted a series of interviews with IPS teams between 2021 and 2023. The interviews mapped the implementation of IPS annually per year across Norway by extracting details on the number of employment specialists in each vocational unit which had been operational between 2012 and 2019. The intensity of IPS (in terms of employment density per 100 000 inhabitants) in the catchment area per year was calculated as a measure of IPS dosage and serves as the exposure in WP2 and WP3.

#### WP2: societal impact of Individual Placement and Support implementation—an effectiveness study

The key objective of WP2 is to measure societal impact of IPS implementation (our independent variable, ie, IPS implementation per catchment area and time period, both as a dichotomy and as the dose of IPS per capita) in terms of employment rates, welfare benefits dependency, healthcare utilisation, crime, victimisation and mortality, and independent living (our dependent variables, data sources listed below). All data will be analysed on an annual basis. Some of our outcomes are categorical (such as mortality), whereas others can be treated as both categorical and continuous variables. Examples of this are employment (which can be categorical—employed/not employed—as well as continuous—as %FTE) and crime (categorical—convicted/not convicted—and continuous—how many criminal acts have been committed). We will carry out our analyses of outcomes both as categorical and continuous variables as appropriate. Additionally, some outcomes are not independent and influence others; an obvious example would be mortality’s influence on all our other outcomes, or if you are receiving a disability pension, you are less likely to become employed.

Using longitudinal registry data and a DID,^67^ we will explore the relative differences in outcomes between where IPS is available and where it is not. Since the rollout of IPS occurred at different times and intensities across Norway, a staggered DID estimation framework will be applied. Staggered treatment occurs when interventions vary in timing across units. The difference in timing will be analysed by year of IPS implementation, as such some participants will be in the control group the whole observation period, whereas others are controls until IPS exposure and in the intervention group after. This approach provides several advantages compared with conventional DID estimation, where the main one is the ability to use both units that have yet to implement IPS and those who never implemented IPS as controls for the analysis. Both the intervention group and the control group will be identified in the Norwegian Patient Registry. The intervention group is defined by residence in an area where IPS has been delivered, which is at about 70 teams in Norway at the end of the observation window 2009–2019. By 2019, IPS was present in all municipalities in Norway. In terms of access to the service, 70.9% of the population lived in an area where IPS was available.^58^

Leaning on data from WP1, we describe how we know when the target population group is potentially exposed to IPS at the psychiatric treatment units. In short, the target group will be defined (per year) by being between 18 and 45 years at inclusion and receiving treatment in public sector specialist mental health services. The cut-off at 45 years old reflects Norwegian policy at the time, as younger employees were initially given priority access to IPS; however, if participants exceed 45 years of age at follow-up, they will be included in the analysis.

The control group is defined as living in a municipality where IPS was not available, and beyond this, by the same criteria as the intervention group. Other forms of employment support will occur in the control group, and we do not have detailed knowledge of the interventions; however, treatment as usual is the common control condition for studies investigating IPS.^22^

As sensitivity analyses, we may also further restrict the sample to a subset with relatively lower income and/or welfare dependency at the time of first contact and not being in full-time education at the time of treatment.

There are an estimated 250 000 people per year receiving mental healthcare in Norway. We assume that 55% of these are in our target group, that is, 82 500 per year. We further assume that 50% of these have been treated in a place where IPS is offered, plus an additional 50% as a control group. There is overlap of individuals over the years, but the extent of this is unclear. Individuals may be re-referred to the health services and thus occur both before and after IPS implementation. They may also move within the country, therefore occurring in different catchment areas at different time points. We assume that in 8 years of recruitment, we have 250 000 unique individuals in the study (given significant overlap between years). It is emphasised that this is an estimate with considerable uncertainty. Main outcomes and definitions for WP2 can be found in table 1.

**Table 1:** Main outcomes and definitions for WP2 societal impact of IPS implementation

Outcome category	Primary outcome	All outcomes and definitions
Employment	Contracted employment (proportion of time)	Contracted employment (dichotomy) is annually recorded (annual weekly work hours) Income (continuous variable, and above threshold for full-time employment) annually recorded Self-employment, both as a dichotomy and based on income
Educational attainment	Ongoing education (proportion of time)	Annually updated information on ongoing education and attainment of degrees at any level
Welfare benefits	Receipt of WAA or DP (proportion of time)	Work assessment allowance (WAA, AAP in Norwegian), both as a dichotomy and period receiving it DP, dichotomy, date of receipt, degree. Sickness absence among employees with an ongoing contract, days as a proportion of contracted workdays
Health	Volume of specialist mental health service utilisation. Proportion of days in active mental specialist treatment, outpatient care counted 1 day per contact, inpatient care counted per day during stay	Volume of use of health services, including both mental and other care, both at specialist level and primary care, and both inpatient and outpatient care
Crime	Any criminal charge or conviction (dichotomy)	Charges and convictions for crimes, dated, and type of crime
Loneliness	Independent living, defined as not receiving support from the municipality for living arrangements, and not living in an institution	Independent living, defined as not receiving benefits for supported living (dichotomy) Marriage and divorce Having own children, living with own children

AAP, Arbeidsavklaringspenger; DP, disability pension; IPS, Individual Placement and Support; WAA, Work Assessment Allowance; WP, work package.

Registry data will be used for the years 2009–2019 for people who meet the specified inclusion criteria. Sources will be linked at the individual level and delivered to the project using encrypted individual identification numbers. Data sources include the following: Control and Payment of Health Reimbursements—operated by the Norwegian Directorate of Health; Norwegian Patient Registry; Historical-Event Database; Cause of Death Registry; National Educational Database; and Norwegian Registry of Offenses (CRIME). Details on contents and purpose in the study can be found in online supplemental table 1.

Using the DID design, we will analyse the overall effectiveness of IPS implementation on four key categories of outcomes:

1. We will explore societal effects of IPS implementation on employment and welfare dependency using registry data for income, tax, employment, contracted work hours, and receipt of work assessment allowance and disability benefits. Educational attainment will be analysed as an independent outcome. We hypothesise increased employment and reduced welfare dependency as a consequence of IPS implementation.
2. We will explore societal effects of IPS implementation on health outcomes using data on contact with specialist mental and non-mental health services, both as inpatient and outpatient, emergency department contacts and hospitalisation for self-harm, general mortality and cause-specific mortality. We hypothesise reduced health service utilisation as a consequence of IPS implementation.
3. We will explore societal effects of IPS implementation on crime rates using data from the Norwegian Registry of Offenses. We hypothesise reduced crime rates as a consequence of IPS implementation.
4. We will explore societal effects of IPS implementation on loneliness using data on independent living, marriage and having children. We hypothesise reduced independent living as a consequence of IPS implementation.

#### WP 3: economic analyses of Individual Placement and Support

The aim of the third WP is to estimate the societal economic consequences of IPS implementation. We will assess whether it is a cost-effective use of resources compared with support as usual from the societal, health and public purse perspectives. To look at the economic impacts of changes over time, including beyond the time period covered by the registry data, we will use an approach known as Markov modelling. These models consist of several mutually exclusive discrete states that an individual could be in during a one cycle of time and cover multiple cycles of time. In our model, there will be four states: actively seeking work, being in employment, losing employment and no longer actively seeking work. The model will also be adjusted to take account of background mortality risk.

Each cycle of time in the model will last 1 year. The probabilities of moving between any one of these states between cycles will be determined using data from WP2, supplemented by information from previous studies and other literature where necessary. These probabilities may also change over time; for example, the probability of lost employment may reduce as individuals spend more cycles of time in employment. Impacts on the use of other services in health, welfare and criminal justice will also be identified from data in WP2.

To determine the resource use and cost of implementing IPS, we will make use of routine administrative data on IPS activity collected in Nordland County. This, for example, includes estimation of the time spent by employment specialists delivering IPS, in addition to supervision, training and other resource costs such as travel. Cost per IPS client reached will then be estimated. Appropriate unit costs will also be attached to the resources impacts on service utilisation, such as health services, welfare and criminal justice. These unit costs will be sourced from multiple published official Norwegian sources. Where official sources are not available, published literature or expert opinion will be used.

The incremental costs incurred for implementation of IPS per change in total number of people employed will be estimated. Administrative data will be used to estimate the distribution of hours worked, as most individuals will only work part time. The number of additional full-time equivalent people employed will then also be estimated. The economic analysis will also take account of the monetary value of changes in utilisation of health services and contact with the criminal justice system.

Cost benefit analyses from the health, public purse and societal perspectives will then be generated from the health, public purse and societal perspectives over short-term and longer-term timeframes. Additionally, we will estimate the return on investment associated with IPS and which sectors benefit from this, for example, what proportion of return on investment accrues to the welfare sector, health sector and criminal justice sectors.

In addition to scenario analyses where different combinations of specific model parameters will be varied, for example, assumptions on cost and resource, likelihood of sustained impacts on employment, a series of univariate and multivariate sensitivity analyses will be conducted, including probabilistic sensitivity analyses to take account of uncertainties in model parameters. Longer-term time periods beyond those available from the registry data will be considered, by modelling scenarios varying assumptions about the persistence of effect and costs of participation in IPS programmes. These longer-term parameters may draw on international long-term evidence from trials and other study designs where available.

All costs will be reported using a single price year, for example, 2025 Norwegian kroner. Costs and outcomes beyond 1 year will be discounted at a rate of 4% per annum using the recommended discount rate for health economic evaluations in Norway.^68^ Discounting is used because in conventional economic theory, costs and benefits in the short term are deemed to have greater value than costs in the future. The discount rate also will be varied in our sensitivity analyses. A Consolidated Health Economic Evaluation Reporting Standards 2022 checklist will also be completed.^69^

### Sample size and power

As this is an effectiveness study, it is not possible to determine the sample size with precision, but we estimate that less than 10% of all people living with SMI in Norway were in contact with specialist psychiatric services during the period 2009–2019. It is also uncertain what effect can be expected, since only a proportion of the target group has been offered IPS, even where IPS has been available.

We have no basis to assume what the effect of IPS should be at the societal level. Furthermore, we have no possibility to make the sample larger, as all of Norway is included, which makes calculations of statistical power redundant.

## Strengths and limitations

Unfortunately, it is a common problem in psychiatry that when implementing clinical procedures with evidence from RCTs, societal effects appear absent. There is sufficient evidence from RCTs for IPS efficacy. This project aims to investigate if effectiveness measured at the level of the society follows IPS implementation. Findings from this project will be key to ascertaining the actual output, or benefit on a societal level, of implementing IPS at scale. The strengths of this study include its design (effectiveness study), which permits investigating the societal footprint of IPS using a large longitudinal cohort. We use registry data without attrition and with high reliability and validity. The exposure to IPS is established by comprehensive interviews and complementary sources. A limitation of the study is related to potential self-report bias in WP1, causing some reliability problems in estimating intensity of IPS in the community. We have limited knowledge of similar interventions and initiatives in the control group. This would introduce bias in favour of the nil hypothesis. We also acknowledge that contextual factors, associated with the IPS intervention areas or not, could bias the analysis. We have limited the observation period to before the COVID-19 pandemic as this is known to influence IPS services.^43^ The many IPS catchment areas and control areas help reduce the risk of contextual factors introducing unobserved confounding. To ascertain IPS programme sustainability, a follow-up with data for 7–10 years beyond this study is needed.

### Patient and public involvement

The IPSRON project is committed to meaningful engagement with people who bring different forms of experience to mental health research. Individuals with lived experience of mental illness are involved in all stages of the project, from planning and implementation to writing and dissemination, ensuring that the research remains relevant to those it seeks to benefit. Their contributions are complemented by insights from carers, mental health professionals, employment specialists and researchers, fostering a collaborative approach that values diverse perspectives.

To support genuine involvement, people with lived experience are actively engaged in meetings, media outreach, educational resource development and presentations at both user-led and academic conferences. In addition, the project contributes to the training of future mental health professionals, social workers and policy makers, ensuring that research findings are effectively translated into practice. By embracing this collaborative and inclusive approach, IPSRON seeks to bridge gaps between research, policy and lived realities, ensuring that findings are accessible, impactful and capable of shaping future mental health services.

### Ethics and dissemination

This is an effectiveness study using registry data. The regional medical research ethics committee has approved the use of registry data without informed consent for this project (approval number 134553). All quantitative data will be stored and analysed using TSD (in Norwegian, Tjenester for Sensitive Data; Services for Sensitive Data in English). TSD is designed for storing and post-processing sensitive data in compliance with the Norwegian ‘Personal Data Act’ and ‘Health Research Act’. TSD is developed and maintained by USIT (in Norwegian, Universitetets Senter for Informasjonsteknologi; the University’s Centre for Information Technology in English) at the University of Oslo and supports research activities run at Norwegian public institutions. Only individuals approved by the ethics committee and the data owners will have access to the data. In the planning of this study, we have followed procedures for data minimisation according to General Data Protection Regulation (GDPR) principles.

The IPSRON project provides a unique opportunity to evaluate the societal impact of large-scale IPS implementation, leveraging Norway’s staggered rollout within a specific timeframe and diverse geographic setting. By linking multiple nationwide registries—covering employment, healthcare, crime, mortality and social indicators—we aim to generate internationally significant evidence using novel, real-world methods. In keeping with this ambition, findings will be submitted to high-impact, peer-reviewed journals, including those with global influence on health and social policy. We will start by analysing employment and welfare benefits as the two primary outcomes, followed by outcome data for criminal charges, healthcare utilisation, mortality and independent living, in the order listed, with publications expected to follow a similar trajectory to the analyses. We aim to publish all main findings in peer-reviewed journals, including nil findings, to ensure transparency, reduce publication bias and contribute to a more accurate and complete evidence base.^37^ We will also disseminate the results directly to stakeholders, to the public through media and our project website, and at relevant conferences and seminars for mental health service providers, policy makers and user organisations. This robust approach, drawing on comprehensive data sources and rigorous analytic methods, has the potential to guide IPS implementation strategies worldwide, inform large-scale policy decisions and ultimately enhance the lives of individuals with moderate to severe mental illness. Empirical studies following this one should study if there is a dose-response association between IPS coverage and effectiveness.

## Supplementary material

10.1136/bmjopen-2025-102179 online supplemental file 1
