# Supplementary material for: Societal consequences of IPS implementation in Norway 2012–2019: study protocol for the IPSRON effectiveness study
Source: BMJ Open. 2026 Mar 6;16(3):e102179. doi: 10.1136/bmjopen-2025-102179 (PMC12970100; doi:10.1136/bmjopen-2025-102179)
Supplement: online supplemental file 1 [file bmjopen-16-3-s001.docx]

**Supplementary Table 1:** Data sources and purpose in the study

| **Data source** | **Content and coverage** | **Coding/ classification** | **Variables used** | **Purpose** |
| --- | --- | --- | --- | --- |
| **Norwegian Patient Registry (NPR)** | Covers all publicly funded inpatient and outpatient treatment in specialist care since 2008 (person-identifiable). | Contact events, per contact, dated.  ICD-10 codes for each contact. | Specialist healthcare contacts, inpatient/outpatient visits, diagnosis. | Indicator of health service use and type of of health problem |
| **Control and Payment of Health Reimbursements (referred to in Norwegian as KUHR)** | Records all billing information from general practitioners, specialists, and outpatient clinics in Norway. Includes fee-for-service tariffs, diagnosis & reason for visit (firm diagnoses and symptom descriptions) | Contact events, per contact, dated.  Diagnostic infoamtion according to ICPC-2 or ICD-10 | Health service use, health worker ID, reimbursement codes | Indicator of health service use. |
| **Information from Statistics Norway** | Longitudinal registry (1992–) with demographic (family type, marital status, children), employment, and social security information (social assistance, sickness benefits, disability pension, work assessment allowance, unemployment benefits) | Event history data, dated. | Employment, sickness/disability benefits, unemployment, work assessment allowance | Longitudinal data on employment, benefit receipt and demographics. |
| **Cause of Death Registry (CDR, referred to as DÅR in Norwegian)** | National registry of all deaths in Norway since 1960. | Event history data, dated. | Date for death, and cause of death coded with ICD-10. | Outcome measures for all-cause mortality, suicide, and non-illness deaths. |
| **National Educational Database (referred to in Norwegian as NUDB)** | Educational attainment and enrolment for all residents. | Annually updated national classification (levels of completed education) | Level of education, current enrolment status | Used as exclusion criterion (enrolled in full-time education) and as outcome (educational attainment). |
| **Norwegian Registry of Offenses (CRIME)** | Data on reported offences, divided into nine main categories (property theft, other profit-making offences, property damage, violence and abuse, sexual offences, drug offences, breach of order and integrity, traffic violations, and other offences) | Event history data, dated.  National offence categories | Reported charges and convictions of crimes. | Measure of criminal behaviour. |
