# Supplementary material for: Societal consequences of IPS implementation in Norway 2012–2019: study protocol for the IPSRON effectiveness study
Source: BMJ Open. 2026 Mar 6;16(3):e102179. doi: 10.1136/bmjopen-2025-102179 (PMC12970100; doi:10.1136/bmjopen-2025-102179)
Supplement: Supplementary file 2 [file bmjopen-2025-102179.reviewer_comments.pdf]

PEER REVIEW HISTORY

BMJ Open publishes all reviews undertaken for accepted manuscripts. Reviewers are asked to complete a checklist review form and are provided with free text boxes to elaborate on their assessment. These free text comments are reproduced below.

ARTICLE DETAILS

Title (Provisional)

Societal consequences of IPS implementation in Norway 2012-2019: study protocol for the IPSRON effectiveness study

Authors

Mykletun, Arnstein; Aars, Nils Abel; Lorentzen, Thomas; Rinaldi, Miles; Sandtorv, Elisabeth; McDaid, David; Moe, Cathrine Fredriksen; Park, A-La; Killackey, Eoin; Brinchmann, B

VERSION 1 - REVIEW

**Reviewer**	**1**
**Name**	**Dawson, Suzanne**
**Affiliation**	**Flinders University, Caring Futures Institute**
**Date**	**11-Jun-2025**
**COI**	**None**

Societal consequences of IPS implementation in Norway 2012-2019: study protocol for the IPSRON effectiveness study

Thank you for the opportunity to review this very impressive and ambitious study. This research extends current knowledge of IPS to examine impacts of the program at a societal level. As such, this study has the potential to have a significant impact internationally for countries advocating for expansion of IPS.

I have only a few suggestions for the authors to consider.

Introduction

Overall, this section is very well written and situates the study and its importance well-in terms of work as a health intervention and IPS specifically as the most effective program. I have a few small suggested edits for the authors to consider.

P4, line 19 ‘when they have a job they see this as a determinant of health and recovery [9, 10]’- I suggest including work as a known social determinant of health, in addition to its importance from the perspective of individuals.

P4, line 22, ‘Work has been an integral part of treatment within mental health services since the late 1700s’- I suggest editing this sentence to emphasise the value of work as treatment for people with mental health conditions, rather than referencing mental health services which would not have remotely resembled contemporary ‘services.’

Please consider more clearly articulating the aims of this study at the end if the Introduction.

Methods

Please provide the timeline for the entire study. Inclusion of a diagram with the 3 discreet studies including the components and how they inter-relate would be helpful for the reader.

Also, please provide details of the investigators to situate them with regards to the research (participants, topic, design etc).

Can the authors expand on why the end date for data collection is at the end of the implementation period (2019) rather than later than this. What resources were provided for implementation? Have these resources changed? How have the programs been sustained? Has there been program drift? These may not be questions that are relevant for this study, but I think that there needs to be some reference to these factors.

A limitation in the design is the lack of exploration of- or mention of program sustainability.

WP1- please state when these interviews were conducted and approximately how many you are anticipating on conducting. Also, a few more sentence on the analysis are needed with more details.

WP2- can the authors please explain why 45 years of age has been selected as the upper age range for people in the study. This does not align with retirement age.

In the section ethics/dissemination- I wondered about emphasising the role that this study will have in advocating for greater uptake of IPS given the relatively low % of people with a SMI that have access to the program- in Norway, and internationally.

**Reviewer**	**2**
**Name**	**Huysmans, Maaike A**
**Affiliation**	**Vrije Universiteit Amsterdam**
**Date**	**18-Jul-2025**
**COI**	**None**

The authors aim to study the effectiveness of real-life IPS implementation on mental health, quality of life and social functioning using registry data on a population level by performing diff-in-diff analyses. Moreover, economics costs will be modelled. The project is ambitious and innovative, but also at times difficult to grasp, most likely because details are missing. Three work packages are proposed, each with its own goal.

Major comments:

• The introduction is rather long. Please consider to shorten it.

• The research question is formulated rather vague, please formulate the research questions more specifically.

• While the diff-in-diff can be used to study effectiveness of real-life IPS implementation on a population level, some assumptions need to be met. The authors did not mention if they will check for these assumptions and how they plan to proceed depending on the outcomes. E.g. previous authors have used a matching procedure. It should at least be considered and mentioned in the protocol paper.

• WP 1; please be more specific how many interviews are planned and how it will be decided when enough people are interviewed? If I understand correctly regional IPS-coordinators will be interviewed? But how many are there and will all be interviewed? Will also other stakeholder be interviewed and how will that be determined? Is this part also submitted for medical ethical approval and is informed consent obtained?

• The authors are still vague in how the implementation measure will be operationalised (for step 2). How are the areas determined, are these the catchment areas? And per area will be determined whether IPS was implemented (yes/no) and the quality of delivery (high/low or more categories, or continuously) based on the fidelity reviews and density of IPS specialist. And how do you link this information to the timeline – per year? I think the authors should provide more information here.

• The authors state that they will use staggered diff-in-diff because the roll-out of IPS occurred at different times and intensities. Do I understand correctly that the analyses will be conducted per month and that over time regions that are first control regions may then over time become an IPS region and also have a higher intensity? Please provide more information on how this works in the analyses, also with these varying levels of intensity.

• At the end of the time window, 70 teams in Norway have implemented IPS. But please provide a reference in this context. In how many areas is that of the total number of areas eg.

• I think the authors should also do an effort to find out what the control employment support is in regions that do not have IPS, in order to correctly interpret the results.

• Do I understand that all individuals eligible in the IPS region will be included in the analyses and also all eligible control individuals?-> ie. Aged 18-45 years and receiving treatment in public sector specialist? And will eligibility be obtained per month?

• P9, line 44 “there is overlap of individuals over the years”, do the authors mean that control individuals become intervention individuals?

• I think the authors could be more specific about the specific variables and categories they will retrieve from the various data sets, at least the most important. It now is rather vague. And again, will they be defined on a monthly basis? E.g. employment – will that be binary (having paid employment > 0 hours per week, or continuously in hours per month?). But also a variable as “crime rates”, mentioned in hypothesis 3; will that be the sum of crimes in the nine categories as mentioned above?

• I suggest making a separate list for defining the independent and dependent variables.

• P11, line 33 -> hypothesis 4; is loneliness operationalised as living independently? I am not sure if loneliness is the right term then.

• P12, line 27; I am not an expert in economic analyses, but as a “normal reader” I am not sure what is the reason for “costs and outcomes are discounted at a rate of 4% per anum” – I would suggest more explanation or context.

• The sample size description does not make much sense to me. I feel that this sample size calculation works differently than in e.g. an RCT. In this design the total population is taken that is eligible. I think it is wise then to check in the first and last years of the study period if the individuals in the intervention and control condition are (too?) unevenly distributed.

• The authors do not mention disturbing factors in the differences between areas, but it would be wise to use the interviews also to collect more information on contextual factors connected to the timeline and the different areas at hand. Eg. Economic or labour market situation. Did the authors consider to also link areas to each other to account for these type of differences that could influence results?

• Which reporting guidelines will be used? I recommend the authors to state this in the protocol, if needed depending on the work package at hand.

• I would recommend authors to further discuss (maybe in the limitations) how certain they are in the end that the effect they try to capture is the effect of IPS? Is there no way in the data to add the registration of an IPS intervention for individuals?

Minor comments:

• Work packages is sometimes abbreviated to WP, but not at all places. Please be consistent.

• P5, line 48. Please provide reference.

• P 7, line 17. Coefficient should be 0.27.

• P 7, line 23. Two decades since…. Has seen? -> I believe this sentence is not correct.

**Reviewer**	**3**
**Name**	**Van Deinse, Tonya B**
**Affiliation**	**The University of North Carolina at Chapel Hill**
**Date**	**08-Aug-2025**
**COI**	**None**

Thank you for the opportunity to review this manuscript, which describes a novel protocol to assess the effectiveness of IPS as implemented in Norway. The protocol proposes using national administrative data to assess population-level effectiveness. Much of the protocol is clear and justified, but some additional attention is necessary in a few areas.

Introduction

For most of the claims made in this section regarding employment and its role within society and among people with mental illnesses, the authors do not locate these claims within a country or region of the world. Is the reader to assume that all trends described reference the Norwegian context? If so, stating this may be helpful for the reader. It is possible that certain claims/trends are not globally applicable, so it may be helpful for the authors to locate their statements within the specific context.

The authors should provide citations for descriptions re: train and place models in the third paragraph in the introduction.

The paragraph that covers people with mental illnesses and criminal justice system involvement is combined with a discussion about cost effectiveness. This paragraph feels a little disjointed and it may be best to either separate the two topics or be clear about how/why they are being discussed together.

Methods

In the section about the setting, the last sentence is referencing young people. However, the discussion about policy reforms focused on people with mental illnesses. Should the reference to young people specify those with mental illnesses? Additionally, is the "challenge" referred to in the last sentence of that section referring to employment among people with mental illnesses? Perhaps just editing for clarity would be helpful here.

Within the sections on mental healthcare, work and welfare, and IPS in Norway, it seems like it would be useful to describe the general implementation of the model within this context. Given the vast differences in both employment resources and mental health services globally, it may be good to orient the reader to how this model has been implemented and generally how it operates. At this stage, it does not appear that this information would be reserved for the findings of this particular study.

This may be a matter of semantics and preference, but perhaps the use of the term comparison group rather than control is more appropriate since the study did not involve randomization.

Discussion of WP1 uses both present and past tense, although it seems this data collection has already occurred. It may be helpful to make this clearer. Additionally, if WP1 was already completed, then it may be good to provide more information about the interviews, the sample, etc. This section also mentions fidelity reviews. The authors should provide more information about the content and process of those reviews. In summary, clarify WP1, especially in terms of whether it is past or future aims, and provide greater detail about the data and the methods used.

For WP2, do the authors have citations for the staggered DID estimation framework?

The intervention and control groups will be identified by the registry, but perhaps the authors can remind the readers that this is data-based identification and not an assignment by an entity.

Is it correct that the study is not using actual enrollment in IPS? In other words, is the assumption that people within a certain age range who have a mental illness would have been exposed to IPS? This seems a little unclear to me as written and it may be helpful to clarify. If this is the case, then more should be written about this in the limitations.

The strengths and limitations section seems to mostly focus on strengths, which is good and appropriate; however, the authors should describe notable limitations to the design of the study (e.g., assumptions about exposure to IPS, if that is the case).

Reviewer **4**

Name **Colantuoni, Elizabeth**

Affiliation **Johns Hopkins University Bloomberg School of Public Health, Biostatistics**

Date **22-Aug-2025**

COI **None**

My main goal was to review the statistical methods in the manuscript, so my comments are generally limited to this. Overall, the statistical assessment is somewhat hampered by the fact that the first phase of the study is underway and the exact number of municipalities that have implemented IPS is unknown and by a lack of specific details on the analysis that is typical in a protocol paper. The pre- and post-IPS implementation target group/sample is defined as adults "18 and 45 years at inclusion and receiving treatment in public sector specialist mental health services". (it is not clear what "at inclusion" means, in 2012? Further, it is not clear if the control group will be the same target group representing all or a fraction of the municipalities that have not implemented IPS. Will adults be included in the

analysis for all years that they satisfy the eligibility definition? There are no specific details about what statistical methods will be used to implement the difference-in-difference estimator and the authors mention the need to account for variation in calendar start time for implementation of IPS across the municipalities, the details on how this will be accounted for in the analysis is not included and no references are provided. If adults can contribute multiple years of data, now will correlation in their outcomes be accounted for. The manuscript could benefit from a table summarizing the primary and secondary outcomes including from what source the data is extracted, the formal definition and how that outcome will be operationalized in the analysis (e.g. continuous variable, binary variable, count). There is no hypothesized effect sizes or justification that the expected sample size will be sufficient to estimate the hypothesized effect sizes in the sample size section. Similar to the analyses for the WP2, the description of the cost analysis for WP3 is vague and no formal details are provided.

VERSION 1 - AUTHOR RESPONSE

Reviewer: 1
Ms. Suzanne Dawson, Flinders University, Southern Adelaide Local Health Network
Comments to the Author:
Societal consequences of IPS implementation in Norway 2012-2019: study protocol for the IPSRON effectiveness study
Thank you for the opportunity to review this very impressive and ambitious study. This research extends current knowledge of IPS to examine impacts of the program at a societal level. As such, this study has the potential to have a significant impact internationally for countries advocating for expansion of IPS.
I have only a few suggestions for the authors to consider.
Introduction
Overall, this section is very well written and situates the study and its importance well-in terms of work as a health intervention and IPS specifically as the most effective program.

Thank you for the encouragement, it is much appreciated.

I have a few small suggested edits for the authors to consider.
P4, line 19 ‘when they have a job they see this as a determinant of health and recovery [9, 10]’- I suggest including work as a known social determinant of health, in addition to its importance from the perspective of individuals.

We have amended accordingly on page 4, and have included a reference (Modini M, Joyce S, Mykletun A, Christensen H, Bryant RA, Mitchell PB, Harvey SB. The mental health benefits of employment: Results of a systematic meta-review. Australas Psychiatry. 2016 Aug;24(4):331-6. doi: 10.1177/1039856215618523. Epub 2016 Jan 15. PMID: 26773063).

P4, line 22, ‘Work has been an integral part of treatment within mental health services since the late 1700s’- I suggest editing this sentence to emphasise the value of work as treatment for people with mental health conditions, rather than referencing mental health services which would not have remotely resembled contemporary ‘services.’

We agree and have revised the sentence (on page 4) to emphasise the value of work as treatment from the perspective of people with mental health conditions and added these references: Ernst, W., *Work, psychiatry and society, c. 1750-2015*. 2016. 1–378; Gammelgaard I, Christensen TN, Eplov LF, Jensen SB, Stenager E, Petersen KS. 'I have potential': Experiences of recovery in the individual placement and support intervention. Int J Soc Psychiatry. 2017;63(5):400-6; and Provencher H, Gregg R, Mead S, Mueser K. The Role of Work in the Recovery of Persons with Psychiatric Disabilities. Psychiatric rehabilitation journal. 2002;26:132-44.

Please consider more clearly articulating the aims of this study at the end if the Introduction.

We have considered this carefully and we have concluded that the aims correspond well with the planned activities in this project.

Methods

Please provide the timeline for the entire study. Inclusion of a diagram with the 3 discreet studies including the components and how they inter-relate would be helpful for the reader.

We have included some further information *in Work packages, data sources and methods* section (page 8) and a schematic overview of the timeline for the work packages is presented in the newly added Figure 1.

Also, please provide details of the investigators to situate them with regards to the research (participants, topic, design etc).

We have added considerable detail to our *Methods and analysis* section (page 6-8) to clarify our design for the reader and avoid any confusion. We hope our added information adequately answers your comment.

Can the authors expand on why the end date for data collection is at the end of the implementation period (2019) rather than later than this.

This is a natural place to stop the study, as the pandemic hampered further roll-out of IPS from 2020, as we have studied in an attached paper (Wittlund, S., Butenko, D., Brandseth, O.L. *et al*. Impact of Covid-19 Restrictions on Individual Placement and Support Service Delivery in Northern Norway. *J. Psychosoc. Rehabil. Ment. Health* **10**, 203–213 (2023). https://doi.org/10.1007/s40737-022-00304-5). Furthermore, after the pandemic, IPS is available in most of Norway, and we no longer have any variation in the data. We have expanded on this on page 6 in the revised manuscript.

What resources were provided for implementation? Have these resources changed?

We have explained further in the revised manuscript that resources for IPS implementation were available upon applications to central government departments, detailed further in the *IPS in Norway* section (page 7&8).

How have the programs been sustained?

We have added details about this in the *IPS in Norway* section (page 7&8) and added references for added details on IPS implementation in Norway (Moe, C., Brinchmann, B., Kolstad, U. *et al.* A qualitative study of the IPS employment specialist role in the context of Nav employment in Norway. *BMC Psychiatry* **25**, 529 (2025). https://doi.org/10.1186/s12888-025-06991-1 and Moe, C., Brinchmann, B., Rasmussen, L. *et al.* Implementing individual placement and support (IPS): the experiences of employment specialists in the early implementation phase of IPS in Northern Norway. The IPSNOR study. *BMC Psychiatry* **21**, 632 (2021). https://doi.org/10.1186/s12888-021-03644-x

Has there been program drift? These may not be questions that are relevant for this study, but I think that there needs to be some reference to these factors.

We have added more information about fidelity to the IPS program in Norway and added the following references in the *IPS in Norway* section (page 8): Bonfils, I. S. (2021). Implementing the Individual Placement and Support approach in institutional settings for employment and mental health services – perceptions and challenges from a case study in Denmark. *European Journal of Social Work*, *25*(3), 471–484; and Proba samfunnsanalyse. (2024). Status for individuell jobbstøtte (IPS) i Norge: Rapport 2024-09 (Proba-rapport nr. 2024-09). ISSN 1891-8093.

A limitation in the design is the lack of exploration of- or mention of program sustainability.

We agree, and we have added a mention that this study needs a follow-up with data for 7 to 10 years beyond this study (on page 12).

WP1- please state when these interviews were conducted and approximately how many you are anticipating on conducting. Also, a few more sentence on the analysis are needed with more details.

The interviews mapped the implementation of IPS annually per year and were conducted between 2021 and 2023 by two members of staff at the Centre for Work and Mental Health. We've added more details to the WP1 section (page 8&9) and added this reference with further details on the process: Aars, N.A.P., et al., *Mapping the Growth of*

Individual Placement and Support Services in Norway. International Journal of Public Health, 2025. Volume 70 - 2025.

WP2- can the authors please explain why 45 years of age has been selected as the upper age range for people in the study. This does not align with retirement age.

The policy in Norway was initially to give priority to younger employees, and thus we made this cut-off for relevance. We have added a sentence on page 10 explaining this in the revised manuscript.

In the section ethics/dissemination- I wondered about emphasising the role that this study will have in advocating for greater uptake of IPS given the relatively low % of people with a SMI that have access to the program- in Norway, and internationally.

The appropriate rate of IPS per capita in a population is not yet determined, and is a question of effectiveness, target groups, organizing and political willingness to prioritize this service. In the empirical studies to follow this one, we will be able to study if there is a dose-response association between IPS coverage and effectiveness. We have added a sentence on page 16 in the revised manuscript.

Reviewer: 2
Dr. Maaike A Huysmans, Vrije Universiteit Amsterdam
Comments to the Author:
The authors aim to study the effectiveness of real-life IPS implementation on mental health, quality of life and social functioning using registry data on a population level by performing diff-in-diff analyses. Moreover, economics costs will be modelled. The project is ambitious and innovative, but also at times difficult to grasp, most likely because details are missing. Three work packages are proposed, each with its own goal.

Major comments:

• The introduction is rather long. Please consider to shorten it.

Other reviewers have commented to the contrary and unfortunately the other reviewers requesting added details have made shortening the introduction a challenge. We hope the revised introduction encompasses added clarity while maintaining readability.

• The research question is formulated rather vague, please formulate the research questions more specifically.

We have added significant detail to the *Study design and setting* section (page 6) and the *Work packages, data sources and methods* section (page 8-14), which hopefully adequately addresses this comment.

• While the diff-in-diff can be used to study effectiveness of real-life IPS implementation on a population level, some assumptions need to be met. The authors did not mention if they will check for these assumptions and how they plan to proceed depending on the outcomes. E.g. previous authors have used a matching procedure. It should at least be considered and mentioned in the protocol paper.

We have clarified the *Sample size and power* section (page 14) significantly and added this reference: Callaway, Brantly, & Sant'Anna, Pedro H. C. (2021). *Difference-in-Differences with Multiple Time Periods. Journal of Econometrics, 225*(2), 200–230.

• WP 1; please be more specific how many interviews are planned and how it will be decided when enough people are interviewed? If I understand correctly regional IPS-coordinators will be interviewed? But how many are there and will all be interviewed?

We've added more details about the interviews in the WP1 section (page 9), and this reference, where even further details can be found: Aars, N.A.P., et al., *Mapping the Growth of Individual Placement and Support Services in Norway.* International Journal of Public Health, 2025. Volume 70 - 2025.

Will also other stakeholder be interviewed and how will that be determined? Is this part also submitted for medical ethical approval and is informed consent obtained?

The different parts of the study is covered by different ethics approvals. We have interviewed several stakeholders, during the implementation process, and this is mentioned in the revised manuscript (page 8) with these references:

Wittlund, S., Butenko, D., Brandseth, O.L. *et al.* Impact of Covid-19 Restrictions on Individual Placement and Support Service Delivery in Northern Norway. *J. Psychosoc. Rehabil. Ment. Health* **10**, 203–213 (2023); Moe, C., Brinchmann, B., Rasmussen, L. *et al.* Implementing individual placement and support (IPS): the experiences of employment specialists in the early implementation phase of IPS in Northern Norway. The IPSNOR study. *BMC Psychiatry* **21**, 632 (2021); Moe, C., Brinchmann, B., Kolstad, U. *et al.* A qualitative study of the IPS employment specialist role in the context of Nav employment in Norway. *BMC Psychiatry* **25**, 529 (2025); Borowska MA, Ørjasæter KB, Borg M, et al. "Without IPS I Think I Would Really Fall Apart": Individual Placement and Support as Experienced by People With Mental Illness—Phenomenological Peer Research Study. *Qualitative Health Research*. 2024;35(8):847-862;
Moe, C., Brinchmann, B., Borg, M., McDaid, D., Rinaldi, M., Killackey, E., & Mykletun, A. (2023). Implementing individual placement and support in Norway. From vocational rehabilitation to an employment scheme. *Social Policy & Administration*, 57(5), 610–625; and Butenko D, Rinaldi M, Moe C, et al. "What I thought was the dream job was a little different than I had expected": A qualitative study

exploring the turnover of IPS employment specialists. *Journal of Vocational Rehabilitation*. 2024;61(1):79-91.

• The authors are still vague in how the implementation measure will be operationalised (for step 2). How are the areas determined, are these the catchment areas? And per area will be determined whether IPS was implemented (yes/no) and the quality of delivery (high/low or more categories, or continuously) based on the fidelity reviews and density of IPS specialist. And how do you link this information to the timeline – per year? I think the authors should provide more information here.

The effect of the implementation is based on catchment areas. We have interviewed stakeholders about when IPS was implemented, how many full-time positions employment specialists were available, the specifics of the catchment area, where the employment specialists were employed, and if there were any restrictions as to target group. This was detailed in a recently published "mapping paper", with annual updates about where IPS was available, measured as the ratio between full time employment specialist positions available per capita in the catchment area (Aars NAP, Brinchmann B, Rinaldi M, Trichet LO, Kolstad U, Steen KS, Moe CF, Lorentzen T, Borg M, McDaid D, Park A-L, Killackey E and Mykletun A (2025) Mapping the Growth of Individual Placement and Support Services in Norway. Int. J. Public Health 70:1608739. doi: 10.3389/ijph.2025.1608739). We have also added more detail on the analysis to our WP2 section (page 9&10). We have updated the revised manuscript with the above details.

• The authors state that they will use staggered diff-in-diff because the roll-out of IPS occurred at different times and intensities. Do I understand correctly that the analyses will be conducted per month and that over time regions that are first control regions may then over time become an IPS region and also have a higher intensity? Please provide more information on how this works in the analyses, also with these varying levels of intensity.

The analysis will be done per year. We have updated the WP2 section in the revised manuscript (page 9) with further details.

• At the end of the time window, 70 teams in Norway have implemented IPS. But please provide a reference in this context. In how many areas is that of the total number of areas eg.

We have added more details (page 8 and 9) and added the following publication for more details: Aars NAP, Brinchmann B, Rinaldi M, Trichet LO, Kolstad U, Steen KS, Moe CF, Lorentzen T, Borg M, McDaid D, Park A-L, Killackey E and Mykletun A (2025) Mapping the Growth of Individual Placement and Support Services in Norway. Int. J. Public Health 70:1608739. doi: 10.3389/ijph.2025.1608739

• I think the authors should also do an effort to find out what the control employment support is in regions that do not have IPS, in order to correctly interpret the results.

Unfortunately, we do not have the resources to interview regions where we assume IPS is not being implemented. As we are rather well connected in the IPS environment in Norway, we believe we have a rather good understanding about where IPS is not being delivered, but we cannot fully guarantee that there in some places may occur IPS-like local initiatives, which are not funded by the normal sources, and also not participating in the national networks for IPS. This will however be a bias in favour of the nil hypothesis and thus a conservative bias. We have added this detail to the revised manuscript.

We have added a reference to that treatment as usual is the common control group in IPS trials on page 10 in the revised manuscript (Brinchmann B, Widding-Havneraas T, Modini M, Rinaldi M, Moe CF, McDaid D, Park AL, Killackey E, Harvey SB, Mykletun A. A meta-regression of the impact of policy on the efficacy of individual placement and support. Acta Psychiatr Scand. 2020 Mar;141(3):206-220. doi: 10.1111/acps.13129. Epub 2019 Dec 19. PMID: 31733146.)

• Do I understand that all individuals eligible in the IPS region will be included in the analyses and also all eligible control individuals?-> ie. Aged 18-45 years and receiving treatment in public sector specialist? And will eligibility be obtained per month?

This is correct, aged 18-45 at the time of contact with public mental health specialist care. Eligibility per year. We have specified this in the revised manuscript (page 10).

• P9, line 44 “there is overlap of individuals over the years”, do the authors mean that control individuals become intervention individuals?

Individuals may be re-referred to the health services, and thus occur both before and after IPS implementation. They may also move within the country, and thus occur in different catchmenta areas at different time points. We have specified this in the re-submitted manuscript (page 9 & 10).

• I think the authors could be more specific about the specific variables and categories they will retrieve from the various data sets, at least the most important. It now is rather vague. And again, will they be defined on a monthly basis? E.g. employment – will that be binary (having paid employment > 0 hours per week, or continuously in hours per month?). But also a variable as “crime rates”, mentioned in hypothesis 3; will that be the sum of crimes in the nine categories as mentioned above?

All data will be analyzed at an annual basis. As to employment and welfare data, we will use both status (if the individual has a job at any time, if the individual is receiving specific welfare benefits at any time) and the monetary values (salary, benefit payments). We have updated the revised manuscript with these details (page 9 & 10).

• I suggest making a separate list for defining the independent and dependent variables.

We have not added separate lists, as some of our outcomes can be both categorical and continuous, however, we have added considerable detail to the WP2 section (page 9 & 10).

• P11, line 33 -> hypothesis 4; is loneliness operationalised as living independently? I am not sure if loneliness is the right term then.

We agree, and we have changed this as advised on page 12 in the revised manuscript.

• P12, line 27; I am not an expert in economic analyses, but as a "normal reader" I am not sure what is the reason for "costs and outcomes are discounted at a rate of 4% per anum" – I would suggest more explanation or context.

This is the recommended procedure, and we have specified this in the revised manuscript (page 12 & 13), along with references to that effect (Hagen, K.P., et al., *Samfunnsøkonomiske analyser*. 2012, Departementenes servicesenter; and Husereau, D., et al., *Consolidated Health Economic Evaluation Reporting Standards 2022 (CHEERS 2022) statement: updated reporting guidance for health economic evaluations*. MDM Policy & Practice, 2022. **7**(1): p. 23814683211061097).

• The sample size description does not make much sense to me. I feel that this sample size calculation works differently than in e.g. an RCT. In this design the total population is taken that is eligible. I think it is wise then to check in the first and last years of the study period if the individuals in the intervention and control condition are (too?) unevenly distributed.

We appreciate the reviewer's comment regarding the assumptions underlying the difference-in-differences design. In our study, we make use of complete population data rather than survey samples, which minimizes the need for sample selection adjustments through matching procedures. As this is a naturalistic effectiveness analysis of the entire country, the sample size is large, but still also not possible for us to influence. We have added a mention of this in the revised manuscript and some further details on our analysis and testing assumptions on page 14.

• The authors do not mention disturbing factors in the differences between areas, but it would be wise to use the interviews also to collect more information on contextual factors connected to the timeline and the different areas at hand. Eg. Economic or labour market situation. Did the authors consider to also link areas to each other to account for these type of differences that could influence results?

We have included a lot of details from the interviews in the WP1 section (page 8 & 9) and added some further details on the relevance of contextual factors in our Strengths and limitations section (page 14 & 15). We do not have any rationale for how to "link" areas as suggested by the reviewer, and this is not a part of our plan.

• Which reporting guidelines will be used? I recommend the authors to state this in the protocol, if needed depending on the work package at hand.

We will start by analyzing employment and welfare benefits as the two primary outcomes. Then we will analyze all data regarding crime, followed by health outcomes, and then the rest. We aim to publish all main findings, including nil findings.We've added further details on page 15 and 16 in the *Ethics and dissemination* section.

• I would recommend authors to further discuss (maybe in the limitations) how certain they are in the end that the effect they try to capture is the effect of IPS? Is there no way in the data to add the registration of an IPS intervention for individuals?

The very idea of the project, as it is approved by the ethics committee, and funded by the Norwegian Research Council, is to examine the societal effect of implementing IPS. In this design, it is actually not important to know who actually received IPS. The very idea is to examine the effect on the society at large. The aim is to study the effectiveness, rather than the efficacy. We've added to our *Study design and setting* section (page 6).

We have used this approach in one publication to date, examining the effectiveness of implementing IPS in one Norwegian municipality compared to some control municipalities (Brinchmann B, Wittlund S, Lorentzen T, Moe C, McDaid D, Killackey E, Rinaldi M, Mykletun A. The societal impact of individual placement and support implementation on employment outcomes for young adults receiving temporary health-related welfare benefits: a difference-in-differences study. Psychol Med. 2024 Jun;54(8):1787-1795. doi: 10.1017/S0033291723003744. Epub 2024 Jan 10. PMID: 38197145.). The project for this protocol is to apply a similar design, though at the scale of the country. The published paper is thus to be regarded as a pilot project in this regard. These details have been added to the revised manuscript.

Minor comments:

• Work packages is sometimes abbreviated to WP, but not at all places. Please be consistent.

Thank you, we have corrected this.

• P5, line 48. Please provide reference.

Thank you, we have corrected this

• P 7, line 17. Coefficient should be 0.27.

Thank you, we have corrected this

• P 7, line 23. Two decades since.... Has seen? -> I believe this sentence is not correct.

Thank you, we have corrected this

Reviewer: 3
Dr. Tonya B Van Deinse, The University of North Carolina at Chapel Hill
Comments to the Author:
Thank you for the opportunity to review this manuscript, which describes a novel protocol to assess the effectiveness of IPS as implemented in Norway. The protocol proposes using national administrative data to assess population-level effectiveness. Much of the protocol is clear and justified, but some additional attention is necessary in a few areas.

We appreciate these encouraging comments.

Introduction
For most of the claims made in this section regarding employment and its role within society and among people with mental illnesses, the authors do not locate these claims within a country or region of the world. Is the reader to assume that all trends described reference the Norwegian context? If so, stating this may be helpful for the reader. It is possible that certain claims/trends are not globally applicable, so it may be helpful for the authors to locate their statements within the specific context.

This is clearly a mistake on our part. The claim that work is good for your health and well-being was first made by Waddel and Burton (Waddell, G. and A. K. Burton (2006). "Is work good for your health and well-being?"), though with rather limited empirical support. Since then, there has been lots of evidence in favour of this claim, though more on the side of well-being on the side of mental health, than hard variables for health. Mechanisms are (somewhat obviously) related to economic benefits of employment, but also likely related to social and psychological aspects, including identity and self-respect, meaning in life, sense of mastery, sense of belonging, sense of security, participation and involvement, and sense of being part of a community. We have amended the manuscript on page 4 and added references (Modini M, Joyce S, Mykletun A, Christensen H, Bryant RA, Mitchell PB, Harvey SB. The mental health benefits of employment: Results of a systematic meta-review. Australas Psychiatry. 2016;24(4):331-6; Rueda S, Chambers L, Wilson M, Mustard C, Rourke SB, Bayoumi A, et al. Association of returning to work with better health in working-aged adults: a systematic review. Am J Public Health. 2012;102(3):541-56; van der Noordt M, H IJ, Droomers M, Proper KI. Health effects of employment: a systematic review of prospective studies. Occup Environ Med. 2014;71(10):730-6; and Axiotidou M, Papakonstantinou D. The meaning of work for people with severe mental illness: a systematic review. Mental Health Review Journal. 2021;26(2):170-9).

The authors should provide citations for descriptions re: train and place models in the third paragraph in the introduction.

We have added clarifying information on page 4 and the following references: Bond, G. R. (2004). Supported employment: Evidence for an evidence-based practice. Psychiatric Rehabilitation Journal, 27(4), 345–359. doi:10.2975/ 27.2004.345.359, and Drake RE, Bond GR. Individual placement and support: history, current status, and future directions. Psychiatry Clin Neurosci Rep. 2023; 2:e122.

The paragraph that covers people with mental illnesses and criminal justice system involvement is combined with a discussion about cost effectiveness. This paragraph feels a little disjointed and it may be best to either separate the two topics or be clear about how/why they are being discussed together.

We agree and have revised accordingly on page 5/6.

Methods
In the section about the setting, the last sentence is referencing young people. However, the discussion about policy reforms focused on people with mental illnesses. Should the reference to young people specify those with mental illnesses? Additionally, is the "challenge" referred to in the last sentence of that section referring to employment among people with mental illnesses? Perhaps just editing for clarity would be helpful here.

Thank you, we have revised accordingly on page 7.

Within the sections on mental healthcare, work and welfare, and IPS in Norway, it seems like it would be useful to describe the general implementation of the model within this context. Given the vast differences in both employment resources and mental health services globally, it may be good to orient the reader to how this model has been implemented and generally how it operates. At this stage, it does not appear that this information would be reserved for the findings of this particular study.

We have added more information and context to the IPS in Norway section (page 7 & 8), and added the following references: Moe, C., Brinchmann, B., Borg, M., McDaid, D., Rinaldi, M., Killackey, E., & Mykletun, A. (2023). Implementing individual placement and support in Norway. From vocational rehabilitation to an employment scheme. Social Policy & Administration, 57(5), 610–625; Aars NAP, Brinchmann B, Rinaldi M, Trichet LO, Kolstad U, Steen KS, Moe CF, Lorentzen T, Borg M, McDaid D, Park A-L, Killackey E and Mykletun A (2025) Mapping the Growth of Individual Placement and Support Services in Norway. Int. J. Public Health 70:1608739. doi: 10.3389/ijph.2025.1608739; and Proba samfunnsanalyse. (2024). Status for individuell jobbstøtte (IPS) i Norge: Rapport 2024-09 (Proba-rapport nr. 2024-09). ISSN 1891-8093.)

This may be a matter of semantics and preference, but perhaps the use of the term comparison group rather than control is more appropriate since the study did not involve randomization.

As this is naturalistic controlled trial, although not randomized, we believe the control group term is the most appropriate, but comparison group may also be applied. As we used the term control group in the pilot study (Brinchmann B, Wittlund S, Lorentzen T, Moe C, McDaid D, Killackey E, et al. The societal impact of individual placement and support implementation on employment outcomes for young adults receiving temporary health-related welfare benefits: a difference-in-differences study. *Psychol Med*. 2024;54(8):1787–95. doi:10.1017/S0033291723003744.), we prefer to keep this term.

Discussion of WP1 uses both present and past tense, although it seems this data collection has already occurred. It may be helpful to make this clearer. Additionally, if WP1 was already completed, then it may be good to provide more information about the interviews, the sample, etc. This section also mentions fidelity reviews. The authors should provide more information about the content and process of those reviews. In summary, clarify WP1, especially in terms of whether it is past or future aims, and provide greater detail about the data and the methods used.

This is correct, WP1 should be past tense. We have revised accordingly.

For WP2, do the authors have citations for the staggered DID estimation framework? The intervention and control groups will be identified by the registry, but perhaps the authors can remind the readers that this is data-based identification and not an assignment by an entity.

We have revised our *Sample size and power* section accordingly (page 14) and added the following reference: Callaway, Brantly, & Sant'Anna, Pedro H. C. (2021). *Difference-in-Differences with Multiple Time Periods. Journal of Econometrics, 225*(2), 200–230.

Is it correct that the study is not using actual enrollment in IPS? In other words, is the assumption that people within a certain age range who have a mental illness would have been exposed to IPS? This seems a little unclear to me as written and it may be helpful to clarify. If this is the case, then more should be written about this in the limitations.

They may have potentially be exposed to IPS, and only a minority will actually be due to patient- and health provider preferences, capacity of the employment specialist, etc. This is not a limitation, but the key feature of the naturalistic trial design and the focus on effectiveness at the societal level rather than efficacy at the level of the individual patient. We have aimed to make this clearer in the revision by adding further detail to the *Study design and setting* section (page 6 onwards).

The strengths and limitations section seems to mostly focus on strengths, which is good and appropriate; however, the authors should describe notable limitations to the design of the study (e.g., assumptions about exposure to IPS, if that is the case).

We disagree that this is a limitation, as outlined above. The main limitation is that we do not clearly know what occurs in the control group, and there may be hints of spillover effects with “IPS-like” initiatives without funding or training by the conventional channels. We believe this is a rather limited problem, but it may not be ruled out. We have made this explicit in the *Strengths and limitations* section (page 14 & 15) in the revised manuscript.

Another limitation is reliability problems from the data collection for the mapping of IPS. It may be limited by memory of the informants and social desirability bias.

We have expanded on this in the revised manuscript (page 14/15.

Reviewer: 4
Dr. Elizabeth Colantuoni, Johns Hopkins University Bloomberg School of Public Health
Comments to the Author:
My main goal was to review the statistical methods in the manuscript, so my comments are generally limited to this. Overall, the statistical assessment is somewhat hampered by the fact that the first phase of the study is underway and the exact number of municipalities that have implemented IPS is unknown and by a lack of specific details on the analysis that is typical in a protocol paper.

We assume this to be a normal challenge related to naturalistic trials, not specific to our project.

The pre- and post-IPS implementation target group/sample is defined as adults "18 and 45 years at inclusion and receiving treatment in public sector specialist mental health services". (it is not clear what "at inclusion" means, in 2012?

Patients can be included over multiple years - the difference in timing will be analysed **by year** of IPS implementation, as such some participants will be in the control group the whole observation period, whereas others are controls until IPS exposure and in the intervention group after. The above details (and more) have been added to the *WP2: Societal impact of IPS implementation: an effectiveness study (page 9 & 10)* and *Sample size and power* sections (page 14).

Further, it is not clear if the control group will be the same target group representing all or a fraction of the municipalities that have not implemented IPS.

It is all, not a fraction. We have expanded on this in the revised MS on page 9 and 10.

Will adults be included in the analysis for all years that they satisfy the eligibility definition?

They will not be excluded if they exceed 45 years of age during follow-up. We have expanded on this in the revised MS on page 10.

There are no specific details about what statistical methods will be used to implement the difference-in-difference estimator and the authors mention the need to account for variation in calendar start time for implementation of IPS across the municipalities, the details on how this will be accounted for in the analysis is not included and no references are provided.

We have added detail on the staggered DID estimation framework to *WP 2: Societal impact of IPS implementation: an effectiveness study* (page 9/10) and *Sample size and power* (page 14) sections and added the following reference: Callaway, Brantly, & Sant'Anna, Pedro H. C. (2021). Difference-in-Differences with Multiple Time Periods. Journal of Econometrics, 225(2), 200–230.

If adults can contribute multiple years of data, now will correlation in their outcomes be accounted for.

The various outcomes are dependent in many ways. For example, receiving disability pension excludes some other welfare benefits, and mortality excludes all. This is restricted by law and regulations, and a lot of it is face valid. Some outcomes are unrelated to all, for example crime. We will account for this in all analyses and have added a mention of this issue in the revised manuscript in the *WP 2: Societal impact of IPS implementation: an effectiveness study* section (page 9 & 10).

The manuscript could benefit from a table summarizing the primary and secondary outcomes including from what source the data is extracted, the formal definition and how that outcome will be operationalized in the analysis (e.g. continuous variable, binary variable, count).

This overlaps with another reviewer's comment. We have indicated this more clearly in the *WP 2: Societal impact of IPS implementation: an effectiveness study* section (page 9 & 10). We would like to keep the option open to use all outcomes as both dichotomies and continuous variables where appropriate, for example, receiving a welfare benefit (dichotomy) and time spent on the benefit (continuous variable). That said, we adhere to openness about findings and approaches attempted.

There is no hypothesized effect sizes or justification that the expected sample size will be sufficient to estimate the hypothesized effect sizes in the sample size section.

This will be the best powered effect analysis of IPS to date, larger than all RCT evidence published, combined. Still, the effect size we aim to measure is modest as this is an effectiveness analysis at the level of the society, rather than efficacy trial. There will likely never be a larger study, as you need both registry data, a mapping of where IPS is implemented, staggered implementation restricted to some but not all catchment areas, and funding for a study at this particular time. Thus, this is an opportunity which warrants a study (and it is consequently funded by RCN for this reason). As there is no

way to make the study larger, we feel that a power calculation is not warranted. We have expanded on this in the *Sample size and power* section on page 14.

Similar to the analyses for the WP2, the description of the cost analysis for WP3 is vague and no formal details are provided.

We agree and have added considerable detail to the WP3 section (page 12 & 13).

Reviewer: 1
If you have selected 'Yes' above, please provide details of any competing interests.: Not applicable

Reviewer: 2
If you have selected 'Yes' above, please provide details of any competing interests.: NA

Reviewer: 3
If you have selected 'Yes' above, please provide details of any competing interests.: Not applicable

Reviewer: 4
If you have selected 'Yes' above, please provide details of any competing interests.: Not applicable

---

VERSION 2 - REVIEW

**Reviewer**	**3**
**Name**	**Van Deinse, Tonya B**
**Affiliation**	**The University of North Carolina at Chapel Hill**
**Date**	**04-Nov-2025**
**COI**	

Thank you for the opportunity to review this revised manuscript. The authors addressed the majority of my comments and questions. The additional context on IPS implementation and WP3 was especially helpful. I only have two more comments to be addressed, both of which I think are necessary for helping readers understand and evaluate the study.

First, there remains some confusion about WP1. The abstract does not refer to WP1 in the past tense, although this is an element of the study that was already completed, based on the authors' responses to reviewers. As stated in the manuscript, WP1 is not an active element of the current study; rather, findings from WP1 (i.e., the implementation of IPS) will be used to inform WP2 and, subsequently, WP3. However, in the manuscript, it is presented

as if it is an aim of the current study. Given that the data collection, analysis, and interpretation of WP1 has already been completed, this is a little confusing. It seems like it would make sense to present WP1 as previous research that informs the current two study activities (i.e., WP2 and WP3). I do think this needs to be clearer and that the abstract should be consistent with the description in the body of the manuscript.

Second, in the response to reviewers, the authors responded to multiple reviewers' feedback by noting that this study is a naturalistic controlled trial but did not clearly identify this in the manuscript (in fact, the term does not appear in the manuscript at all). Given that multiple reviewers had questions about the design, it is safe to assume other readers may as well. Consequently, in the methods section, it would be helpful to describe naturalist controlled trials and provide citations so a future reader can have additional context. This may address readers' questions ahead of time as they consider potential study limitations (e.g., sample, effect size, exposure to the intervention, etc.) similar to the reviewers' comments. Additionally, this design should also be mentioned in the abstract.

Thank you again for the opportunity to review this innovative study.

Reviewer **4**

Name **Colantuoni, Elizabeth**

Affiliation **Johns Hopkins University Bloomberg School of Public Health, Biostatistics**

Date **21-Oct-2025**

COI

The authors have responded to my questions/comments for the most part and the manuscript has more detail related to the methods, especially wrt WP3. I maintain my suggestion that the paper would benefit from a table, including it as a supplement would work, where the authors provide precise definitions for outcomes for WP2. The added text detailing the difference-in-difference assumptions that is currently included in the sample size and power calculation section is best moved to the statistical analysis section as this really is not related to sample size/power but to the key assumptions that underpin the analytic plan. The revised manuscript is significantly longer than the first draft; the word count could be reduced without losing core content/concepts by reducing the length of the introduction and moving some details in various sections of the methods to supplement.

VERSION 2 - AUTHOR RESPONSE

Reviewer: 3
Dr. Tonya B Van Deinse, The University of North Carolina at Chapel Hill
Comments to the Author:

Thank you for the opportunity to review this revised manuscript. The authors addressed the majority of my comments and questions. The additional context on IPS implementation and WP3 was especially helpful. I only have two more comments to be addressed, both of which I think are necessary for helping readers understand and evaluate the study.

First, there remains some confusion about WP1. The abstract does not refer to WP1 in the past tense, although this is an element of the study that was already completed, based on the authors' responses to reviewers.

Thank you for pointing this out, we have now corrected this error in the abstract.

As stated in the manuscript, WP1 is not an active element of the current study; rather, findings from WP1 (i.e., the implementation of IPS) will be used to inform WP2 and, subsequently, WP3. However, in the manuscript, it is presented as if it is an aim of the current study. Given that the data collection, analysis, and interpretation of WP1 has already been completed, this is a little confusing. It seems like it would make sense to present WP1 as previous research that informs the current two study activities (i.e., WP2 and WP3). I do think this needs to be clearer and that the abstract should be consistent with the description in the body of the manuscript.

Thank you, we have changed the way the section on WP1 is written (page 8), as well as removing details on the procedure and instead referring to our previous publication on our work in WP1.

Second, in the response to reviewers, the authors responded to multiple reviewers' feedback by noting that this study is a naturalistic controlled trial but did not clearly identify this in the manuscript (in fact, the term does not appear in the manuscript at all). Given that multiple reviewers had questions about the design, it is safe to assume other readers may as well. Consequently, in the methods section, it would be helpful to describe naturalist controlled trials and provide citations so a future reader can have additional context. This may address readers' questions ahead of time as they consider potential study limitations (e.g., sample, effect size, exposure to the intervention, etc.) similar to the reviewers' comments.

Thank you, we have added a paragraph in Study design and setting (page5/6) describing naturalistic trials, along with references pertaining to the research design.

Additionally, this design should also be mentioned in the abstract.

Thank you, we have added a sentence about this to the abstract.

Thank you again for the opportunity to review this innovative study.

Reviewer: 4
Dr. Elizabeth Colantuoni, Johns Hopkins University Bloomberg School of Public Health
Comments to the Author:
The authors have responded to my questions/comments for the most part and the manuscript has more detail related to the methods, especially wrt WP3. I maintain my suggestion that the paper would benefit from a table, including it as a supplement would work, where the authors provide precise definitions for outcomes for WP2.

We have added Table 1 (Main outcomes and definitions for WP2 Societal impact of IPS implementation) to page 10/11.

The added text detailing the difference-in-difference assumptions that is currently included in the sample size and power calculation section is best moved to the statistical analysis section as this really is not related to sample size/power but to the key assumptions that underpin the analytic plan.

This section has been moved to the methods and analysis section (Work packages, data sources and methods) on page 8.

The revised manuscript is significantly longer than the first draft; the word count could be reduced without losing core content/concepts by reducing the length of the introduction and moving some details in various sections of the methods to supplement.

We have significantly reduced the length of the introduction and moved Data sources, contents and purpose from the Methods and analysis section to Supplementary Table 1.

VERSION 3 - REVIEW

**Reviewer**	**3**
**Name**	**Van Deinse, Tonya B**
**Affiliation**	**The University of North Carolina at Chapel Hill**
**Date**	**02-Dec-2025**
**COI**	

The authors have addressed all comments from this reviewer. This is a very interesting study, and I look forward to reading the results.

**Reviewer**	**4**
**Name**	**Colantuoni, Elizabeth**

Affiliation **Johns Hopkins University Bloomberg School of Public Health, Biostatistics**

Date **30-Nov-2025**

COI

I appreciate the opportunity to review the revised manuscript. The authors have now responded to all my comments and I have no further requests.
